# The biomechanical effects of foraminoplasty of different areas under lumbar percutaneous endoscopy on intervertebral discs: A 3D finite element analysis

**DOI:** 10.1097/MD.0000000000019847

**Published:** 2020-04-24

**Authors:** YiZhou Xie, Qun Zhou, Xinling Wang, Qiang Jian, Xiaohong Fan, Yang Yu, Dangwei Gu, WeiDong Wu

**Affiliations:** a Hospital of Chengdu University of Traditional Chinese Medicine; b Chengdu University of Traditional Chinese Medicine, Chengdu, Sichuan Province; c Southern Medical University, Guangzhou, Guangdong Province, P.R. China.

**Keywords:** 3D finite element analysis, biomechanical, foraminoplasty, lumbar percutaneous endoscopy

## Abstract

**Background:**

We set out to evaluate the biomechanical influence of foraminoplasty on intervertebral discs in different areas under lumber percutaneous endoscopy through the use of a three-dimensional finite element.

**Methods:**

We established a normal 3D finite element mode of L_3–5_, using simulate lumbar percutaneous endoscopy by carrying out cylindrical excision of a bone whose diameter was 7.5 mm on the L_5_ superior articular process and the L_4_ inferior articular process, respectively. We therefore obtained 3 models. The first was the normal lumbar model, the second the L_4_ inferior articular process shaped model, while the third was the L_5_ superior articular process shaped model. We compared the biomechanics of discs of L_3/4_ and L_4/5_ in states of forward flexion, backward extension, left and right flexion as well as left and right rotation.

**Results:**

When the L_4_ inferior articular process shaped model was in backward extension, left rotation, and right rotation, the stress of the L_4/5_ disc was greater than in the normal model, especially in the state of extension. When the L_5_ superior articular process shaped model was in left and right rotation, the biggest stress of the L_4/5_ disc increased slightly. However, no matter which way the L_5_ superior articular process or the L_4_ inferior articular process of model was shaped, the stress impact of the L_3/4_ disc was small.

**Conclusions:**

There is more biomechanical influence on the L_4/5_ disc when carrying out a foraminoplasty on L_4_ inferior articular process under a lumber percutaneous endoscopy. In addition, the influence of both types of surgery on the stress of L_3/4_ disc is small.

## 1 Introduction

In recent years, percutaneous endoscopy has been widely used for treating degenerative diseases of the spine.^[1]^ As technological grows and surgical equipment is upgraded, indications for this kind of surgery have extended from lumbar disc herniation to lumbar spinal stenosis, while the surgical approach has shifted from the transforaminal lateral posterior approach to the posterior interlaminar approach.^[2]^ It is well-known that L_4/5_ is the most common segment of lumbar disc herniation. During L_4/5_ lumbar percutaneous endoscopic surgery under a lateral posterior approach, the facet joint of L_4/5_ is the main obstacle preventing the working channel from entering the anterior space of the dural sac in the spinal canal. In contrast, in the posterior approach, the narrowing of the L4/5 lamina space obstructs the placement of the working passage into the spinal canal.

Therefore, the technique of foraminoplasty is the most critical and crucial step in the 2 approaches, and is also a prerequisite for successful surgery. However different methods of foraminoplasty could have distinct mechanical influences on the lumbar intervertebral disc, based on the anatomical characteristics of the facet joint. It is also worth considering despite a large number of studies, few authors have mentioned that changes in the stress effect of intervertebral foramen shaping surgery methods and paths on the disc would change the influence on the disc.^[3–5]^ In this study, a three-dimensional finite element analysis was utilized to analyze the impact of L_5_ superior articular process shaping and L_4_ inferior articular process shaping, respectively, on changes in the stress data of L_4/5_ and L_3/4_ intervertebral discs affected after the surgery, when the 2 endoscopic approaches of lateral posterior and posterior approaches were used.

## 2 Methods

### 2.1 General data

The participant is a healthy adult male, aged 25, weight 65 kg, height 170 cm. He was free from lumbar deformity, disc herniation, degenerative diseases, and other diseases following lumbar digital radiography (DR), computed tomography (CT), and magnetic resonance imaging (MRI) examination. Before the study, the participant gave informed consent, which was reviewed by the Institutional Review Board (IRB) of the authors’ affiliated institutions. This research has been approved by the IRB of the authors’ affiliated institutions.

### 2.2 Methods

#### 2.2.1 Software and equipment

Equipment used included Siemens Somatom Sensation 64 row helical CT (supported by the department of radiology, Hospital of Chengdu University of Traditional Chinese Medicine); Mimics 16.0, Hospital of CDUTCM (professional medical image application software); Creo3.0 (surface design professional software); Geomagic Studio 12.0 (3D modeling reverse engineering software); and ANSYS15.0 (finite element analysis software). All the above experimental software was provided by the key laboratory of biomechanics of Southern Medical University.

#### 2.2.2 L3–5 3D finite element modeling

**Figure 1:**
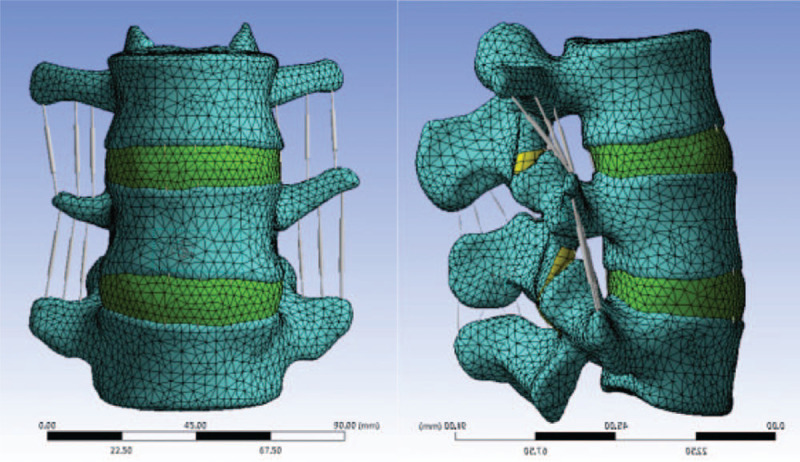
The finite element model of L3–5 (M1) is established by scanning the lumbar of a 30-year-old young male volunteer through Siemens Somatom Sensation 64 multi-sliced spiral CT (MSCT) and constructing using ANSYS and MIMICS software.

The participant's L_3–5_ was scanned by Siemens Somatom Sensation 64 row helical CT with a thickness of 0.625 mm. The obtained two-dimensional image was saved in DICOM format. All obtained DICOM graphics were imported into Mimics 16.0, after which the software was run to build the L_3–5_ three-dimensional image model with the support of Creo3.0 and Geomagic Studio 12.0. After polishing and other modifications, the preliminary model was transferred into ANSYS to perform subsequent network division and obtain the bony finite element model.^[6]^ Based on typical physiological structure, an anterior longitudinal ligament, posterior longitudinal ligament, interspinous ligament, supraspinous ligament, intertransverse ligament, ligamenta flava, and intervertebral disc were added to the model. A typical L_3–5_ three-dimensional finite element model was constructed (see Fig. 1), and normal values were given to the parameters of each structure of the obtained model (see Table 1).^[6]^ For the start and end points as well as the transverse protrusion areas of the model, values were assigned according to normal anatomical relations,^[7,8]^ while all joint surfaces were defined as a sliding contact and the friction coefficient between joint surfaces was set at 0.1.^[9]^

**Table 1:**
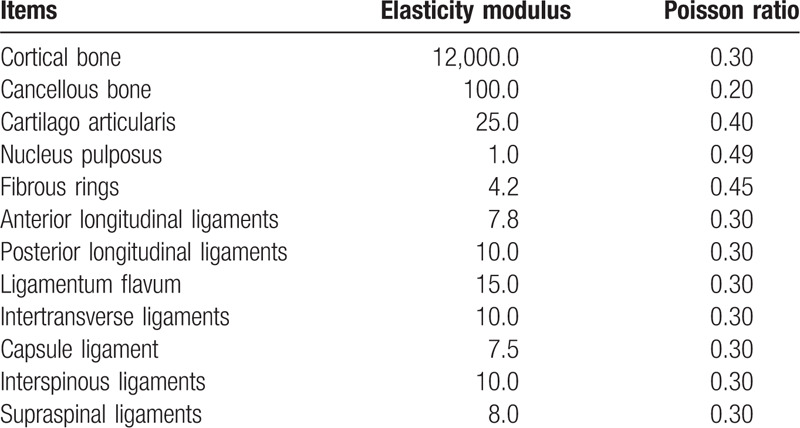
Finite element model material properties.

#### 2.2.3 Verifying the validity of the model

The finite element model obtained in this study was compared and analyzed with various data obtained by autopsy, including SHIM.^[10]^ Comparisons were conducted under the same environment, condition constraints and load, and with the full range of activities in all directions. Additionally, ligament data at each position were modified to ensure model data was within the range of biomechanical data obtained by anatomy such as SHIM, hence ensuring the effectiveness and reliability of modeling (see Table 2).

**Table 2:**
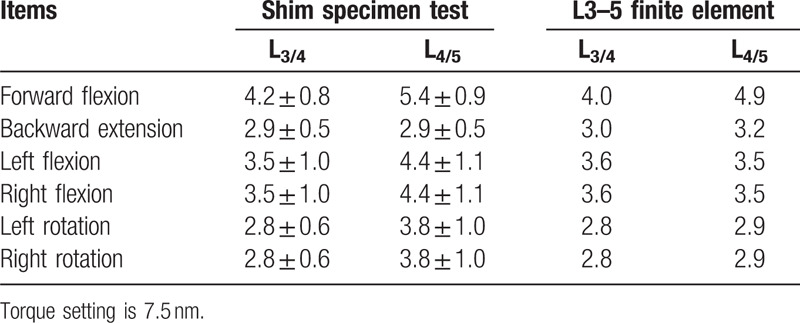
Model verification results (*×* ± *s*, °).

#### 2.2.4 The model of articular process shaping was constructed

**Figure 2:**
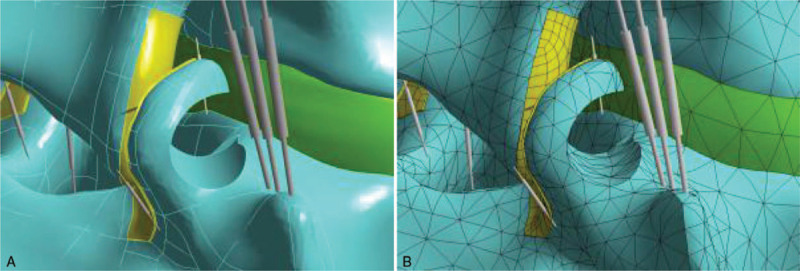
A. Three-dimensional finite element model after L5 superior articular process foraminoplasty. B. Meshed three-dimensional finite element model after L5 superior articular process foraminoplasty.

**Figure 3:**
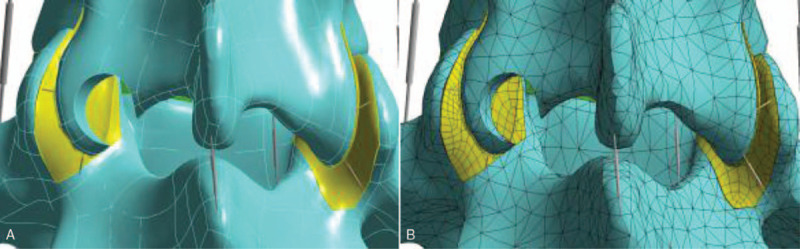
A. Three-dimensional finite element model after L4 inferior articular process foraminoplasty. B. Meshed three-dimensional finite element model after L4 inferior articular process foraminoplasty.

Based on the above finite element model, we simulated transdermal endoscopic surgery by selecting the left superior articular process of L_5_ as the puncture point through the lateral posterior approach and developed a precise surgical guidance process. A working channel was maintained at an angle of 30° to the coronal plane to remove the left superior articular process of L_5_ (*d* = 7.5 mm). When the posterior approach was selected for simulated surgery, the puncture point was selected as the inferior articular process of L_4_ and s precise surgical guidance route was developed. The left inferior articular process of L_4_ was then excised (*d* = 7.5 mm). After that, the L_5_ superior articular process shaped model and the L_4_ inferior articular process shaped model on the left of the body could be obtained, respectively, to perform subsequent experiments (see Figs. 2 and 3).

#### 2.2.5 Boundary and load

The degree of freedom of the inferior surface of the L_5_ vertebra was set to 0, and 400 N pressure was vertically applied to the endplate on the superior surface of the L_3_ vertebra, thus fully reflecting the lumbar bearing condition of healthy people when standing vertically. Following this, the pure torque of 7.5 Nm was applied in the forward flexion, backward extension, left and right flexion, and left and right rotation directions respectively. The load was then divided into 6 motion states: forward flexion, backward extension, left and right rotation, and left and right lateral flexion. Surgical effects on the stress parameters of the corresponding and adjacent intervertebral discs were compared with parameters of the constructed normal model.

## 3 Results

**Figure 4:**
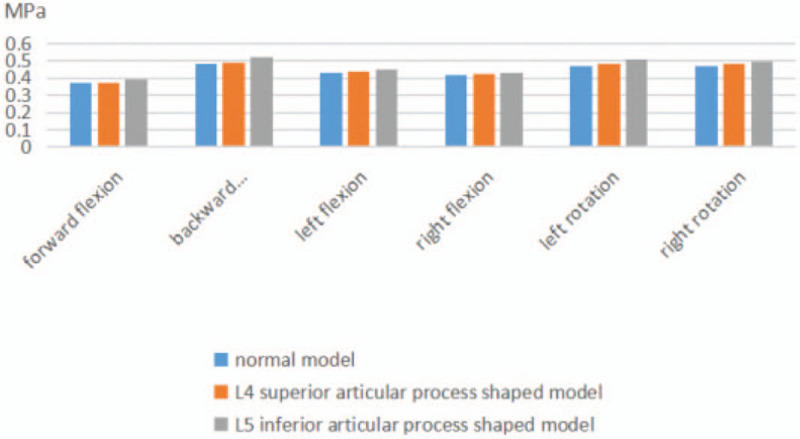
Maximum stress of L4/5 intervertebral disc after 2 forming methods.

Stress values of the L_4/5_ disc measured in the model of L_4_ inferior articular process shaped were, 0.375 and 0.490 MPa, 0.440 and 0.423 MPa, 0.482 and 0.478 MPa, when the model was in forward flexion and backward extension, left and right lateral flexion, and left and right rotation. Stress increased significantly when the position was rotated to left or to right. The stress values of the L_4/5_ intervertebral disc (0.390 and 0.520 MPa, 0.450 and 0.430 MPa, 0.510 and 0.498 MPa) were measured in the model of L_5_ superior articular process shaped in forward flexion and backward extension, left and right lateral flexion, and left and right rotation. Stress increased significantly when the position was in backward extension or rotation (see Fig. 4).

**Figure 5:**
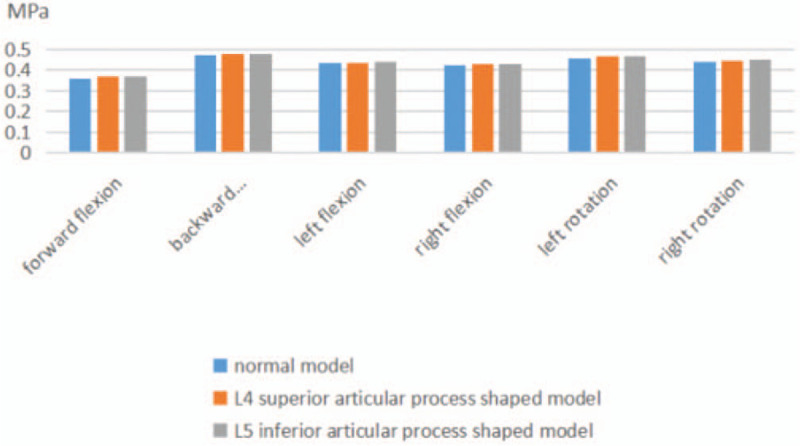
Maximum stress of L3/4 intervertebral disc after 2 forming modes.

Following that, when the model of L_4_ superior articular process shaped was in forward flexion and backward extension, left and right lateral flexion, and left and right rotation, the stress value of the L_3/4_ intervertebral disc was, 0.425 and 0.462 MPa, 0.368 and 0.478 MPa, 0.436 and 0.454 MPa respectively. When the model of the L_5_ superior articular process shaped was in forward flexion and backward extension, left and right lateral flexion, and left and right rotation, the stress value of L_3/4_ intervertebral disc was, 0.437 and 0.426 MPa, 0.369 and 0.480 MPa, 0.461 and 0.452 MPa respectively (see Fig. 5 for details).

## 4 Discussion

### 4.1 Research status of biomechanics of lumbar transdermal endoscopy

It has long been thought that traditional posterior direct decompression or assisted fusion was the most effective treatment for both lumbar spinal stenosis and lumbar disc herniation.^[11,12]^ However, the great damage this treatment causes, including damage to the posterior column of the spine, the formation of a scar around the nerve, and the risk of anesthesia have been criticized by experts around the world. Lumbar percutaneous endoscopy is a minimally invasive technique which has low trauma and a rapid recovery as well as being relatively low cost, and offering a relatively good spinal stability protection. This technique has attracted global interest and been accepted by a growing number of patients with lumbar spine diseases.^[13–16]^ Since this technology was developed, various technical schools and academics have offered their opinions about it.^[2]^ In fact, the diversity of this technology is mainly reflected in the process of foraminoplasty before the working channel enters the spinal canal. Particularly for the L_4/5_ segment, this surgery has 2 widely used methods: L_5_ superior articular process shaping and L_4_ inferior articular process shaping through the lateral and posterior approach.^[17–19]^ However, despite numerous scientific studies,^[3–5]^ there is a lack of research into the influence of these 2 surgical approaches on the degeneration of the corresponding and adjacent segments of the disc after surgery is complete. We set out to fill that gap in the literature.

A circular saw with an external diameter of 7.5 mm was applied to the superior articular process of L_5_ and the inferior articular process of L_4_ respectively, through the lateral posterior approach, to remove the cylindrical bone area with diameter of 7.5 mm. 400 N pressure was then applied on the superior surface of the L_3_ centrum. Stress changes and influences on the discs of L_3/4_ and L_4/5_ were analyzed in 6 motion states.

### 4.2 Three-dimensional finite element analysis of the changes in disc stress caused by different approaches to facet arthroplasty

Respectively, the normal L_4_ inferior articular process shaped and L_5_ superior articular process shaped models constrained on inferior surface and the lateral stresses in different directions were applied under axial loads. Following this, we analyzed stress parameter changes to the intervertebral discs L_3/4_ and L_4/5_ under forward flexion, backward extension, left lateral flexion, right lateral flexion, left rotation, and right rotation. Significant changes of the L_4/5_ intervertebral disc in the L_4_ inferior articular process shaped model mainly appeared in left and right rotations (0.482, 0.478 MPa) which are 5.60% and 3.76% larger than in the normal model. For the L_5_ superior articular process shaped model, the maximum stresses on the L_4/5_ intervertebral disc were 0.520, 0.510, and 0.498 MPa under backward extension, left rotation, and right rotation respectively. These were 10.57%, 10.78%, and 7.63% higher than those of the normal lumbar model. This indicates that the postoperative model constructed at different parts and paths has the most significant changes for biomechanic values under the rotation of the corresponding lumbar segments affected, especially under the circumstance of the L_5_ superior articular process shaped. Additionally, when the superior articular process of L_4_ is partly excised, backward extension will also cause a significant increase in stress on the L_4/5_ disc.

When the L_4_ superior articular process shaped model was in forward flexion and backward extension, right and left lateral flexion, and left and right rotation, the stress values of the L_3/4_ intervertebral disc were 0.368 and 0.478 MPa, 0.436 and 0.430 MPa, 0.465 and 0.444 MPa respectively. When the L_5_ inferior articular process shaped model was in right and left lateral flexion, forward flexion and backward extension, and left and right rotation, stress values were 0.369 and 0.480 MPa, 0.442 and 0.432 MPa, 0.468 and 0.452 MPa respectively. It can be seen that there is no significant difference between the L_3/4_ stress change and the normal model under the 6 activity conditions after the 2 surgical approaches. This suggests that lumbar transcutaneous endoscopic foraminoplasty in any area has little effect on the biomechanics of adjacent segments.

By analyzing lumbar anatomy, we know that its superior articular process originates from the pedicle and lamina junction, yet its articular surface is concave, facing the posteromedial side, and its articular surface is wider than the inferior articular process. The inferior articular process of the lumbar is an extension of the lamina, with the articular surface pointing outward. Facet joints are firmly locked together in a manner close to the tenon and the mortise.^[20]^ This arrangement of the lumbar spine limits its rotation and translation. When we conduct a foraminoplasty, this motion limitation is changed, resulting in increased stress of the corresponding segment of the intervertebral disc during rotation. However, the concave and broad articular surface of the inferior articular process of L_5_ was not damaged during the cutting of the inferior articular process of L_4_, meaning the remaining section of the inferior articular process of L_4_ could still be locked by the articular surface of the superior articular process of L_5_ at a certain extent after the shaping. Therefore, the increase of its biomechanics was significantly less than that of L_5_ superior articular process shaping. Nevertheless, the biomechanics of L_3/4_ intervertebral disc were almost unaffected as L_4_ superior and L_3_ inferior articular processes were not ground down.

## 5 Conclusion

In summary, the L_3–5_ lumbar spine model established by the three-dimensional finite element technique in this study has an intuitive, vivid geometric shape, and the 2 facet shaping techniques based on this model can almost completely compare with clinical practice. In a simulation of the mechanical effect of human body weight, through the mechanical analysis under 6 motion states, we found that the L_5_ superior articular process shaping in the lateral posterior approach as well as the L_4_ inferior articular process shaping in the posterior approach developed significant biomechanical changes on the L_4/5_ disc, especially the L_5_ inferior articular process shaping in the posterior approach. In contrast, the 2 foraminoplasty methods had no significant influence on biomechanics of the adjacent segment L_3/4_ disc.

## Author contributions

Yizhou Xie, Wang Xinling, Yang Yu, Qun Zhou, Xiaohong Fan, and Weidong Wu performed the experiments. Yizhou Xie, Qun Zhou, Xinling Wang, and Yang Yu wrote the paper. Yizhou Xie, Yang Yu, Xiaohong Fan, Dangwei Gu, Weidong Wu, and Qiang Jian reviewed Edited the manuscript. All authors read and approved the manuscript.
